# Do pro-environmental factors influence sustainable behavior? The moderating role of climate change concern in China and Pakistan

**DOI:** 10.3389/fpsyg.2026.1704513

**Published:** 2026-03-11

**Authors:** Zahida Iqbal, Liu Youjin, Haroon Imtiaz, Najid Ahmad

**Affiliations:** 1 School of Business, Hunan University of Science and Technology, Xiangtan, China; 2 Department of Mechanical Engineering, NUST College of Electrical & Mechanical Engineering, National University of Sciences & Technology, Islamabad, Pakistan

**Keywords:** energy saving, single-use plastics, sustainable behavior, Theory of Planned Behavior, waste sorting management

## Abstract

This study examines the factors driving pro-environmental behavior by focusing on energy-saving, waste sorting, and reducing single-use plastics. Utilizing 380 participants' survey data from China and Pakistan, our study considers the Theory of Planned Behavior to examine attitudes, subjective norms, perceived behavioral control, and climate change concern in shaping environmental intentions. Employing a structural equation modeling, we find that intentions related to climate change were positive and significantly associated with all three targeted behaviors, i.e., energy saving, waste sorting, and single-use plastic behavior. Across both countries, attitudes, perceived behavioral control, subjective norms, and climate change concern are significant predictors of pro-environmental intention. Country-specific analysis reveals that attitudes and climate change concern in China significantly predict environmental intention, whereas in Pakistan, environmental intentions significantly predict single-use plastic use behavior. Moderation analysis shows that climate change concern did not significantly moderate the relation between intention to take climate action and waste sorting behavior for China, whereas it substantially moderates for Pakistan. However, single-use plastic reduction and climate change concern significantly moderate the relationship between intention and climate change concern and single-use plastic reduction for both countries. The findings highlight the evolving adoption of eco-friendly practices and emphasize the need for environmental policies to enhance pro-environmental behavior.

## 1 Introduction

The increasing concerns about rapid climate change, resource depletion, and environmental degradation (Warneke et al., 2023; Kirikkaleli et al., 2023) urge us to focus on the rise of global warming and climate change to work jointly to reverse the situation before it turns into a serious threat to humanity on the earth. Considering that climate change poses a significant threat to humanity (Obradovich et al., 2018), mitigating its effects through sustainable behaviors (SBs) is essential (Meet et al., 2024). Faghani et al. (2023) observed that human activities, driven by unsustainable behaviors ranging from dietary habits and food waste to energy use, transportation preferences, and consumption patterns, lead to environmental degradation. These activities contribute to pollution emissions and biodiversity loss, with far-reaching consequences, including climate change and natural disasters (Teraa and Bencherif, 2022; Wang et al., 2021; Kaya Kanli and Küçükefe, 2023).

Recent research pointed out that pro-environmental behavior plays a key role in improving the environment (He et al., 2023), such as adopting pro-environmental practices, and individuals helping mitigate the damage caused by unsustainable actions (Dietz et al., 2009). The sustainable practices are often referred to as “pro-environmental behaviors,” “green behaviors,” or sustainable actions (Batool et al., 2024; Fu et al., 2017). Indeed, pro-environmental behaviors (PEBs) encompass actions aimed at reducing environmental harm or improving living conditions through sustainable practices (Ogiemwonyi and Jan, 2023). It has been highlighted that public engagement in PEBs, such as energy conservation (Ogiemwonyi et al., 2020; Zhang et al., 2020), waste sorting (Vorobeva et al., 2023), reducing single-use plastics (Oludoye et al., 2024), and adopting low-carbon lifestyles (Ma and Chen, 2025) is vital for achieving global climate targets (Gulliver et al., 2021). Given this, fostering sustainable behavior at the individual level emerged as a vital strategy to fight against climate change and lead to environmental progress.

Using surveyed data obtained through an online questionnaire from China and Pakistan, our study aims to investigate three key pro-environmental behaviors—energy-saving practices, plastic waste reduction, and waste sorting behavior. These behaviors are essential for environmental correction urgencies for both countries (Wang et al., 2021; Lin and Jia, 2023; Nawaz et al., 2022; Tan et al., 2023), which are well aligned with the United Nations Sustainable Development Goals (SDGs) established in 2015 for the 2030 agenda. Specifically, promoting energy-saving supports SDG 7 (affordable and clean energy), which targets doubling the global rate of energy efficiency improvements by 2030. Effective waste management through sorting and recycling contributes to SDG 12, which is responsible consumption and production, particularly target 12.5, which aims to reduce waste generation substantially by 2030. Finally, reducing single-use plastics addresses SDG 14 (life below water), targeting a significant reduction in marine pollution by 2030 (United Nations, 2015). By focusing on these behaviors, the study responds to the pressing environmental challenges faced by both countries and focuses on advancing global sustainability commitments. The choice of China and Pakistan is inspired by the fact that both are developing nations, working together for the China-Pakistan Economic Corridor (CPEC) to fight against the common issues, such as climate change and global warming, and to foster faster innovation. Further, both countries face environmental challenges that require joint efforts to work for sustainability.

This research has three main objectives:

(1) To examine the effect of key Theory of Planned Behavior constructs-attitude toward climate change, subjective norms, perceived behavioral control, and climate change concern on individuals' intentions to engage in climate change mitigation across China and Pakistan.
(2) To investigate how climate change intention translates into three critical pro-environmental behaviors, energy saving, waste sorting, and single-use plastic reduction-using both pooled and country-specific samples.
(3) To assess the moderating role of climate change concerns in moderating the relationship between climate change intentions and actual pro-environmental behaviors, highlighting the influence of cultural, institutional, and country-specific contexts in China and Pakistan.

This study contributes to the literature by simultaneously investigating three prominent pro-environmental behaviors in Pakistan and China: energy conservation, single-use plastic reduction, and waste sorting. In doing so, we consider the aggregated sample of China and Pakistan, and individual country analyses were carried out for better sustainability policy suggestions. We rely on surveyed data obtained through an online questionnaire from 380 participants to extract facts. We employed the Theory of Planned Behavior (TPB) across multiple pro-environmental behaviors, considering its advantages in the form of a comprehensive structure, ease of variable measurement, and its capacity to integrate personal, social, and contextual factors. Notably, existing studies focus on a single behavior over time, while we consider a more holistic approach by simultaneously examining three key behaviors— saving energy, reducing single-use plastics, and waste sorting across China and Pakistan, and analyzing the aggregated sample as well as the country-level analysis that's important for policy.

The rest of the paper is structured as follows: Section 2 offers a literature review and builds research hypotheses. Section 3 outlines data details and the methodological approach. Section 4 reports the empirical findings, followed by a discussion in Section 5. Section 6 concludes the paper with the policy suggestions.

## 2 Literature review and hypotheses development

The Theory of Planned Behavior (TPB) is a critical established framework for predicting and understanding human behavior. At its core, TPB posits that an individual's behavior is primarily influenced by three key components: attitudes toward the behavior, perceived behavioral control, and subjective norms, all of which shape behavioral intentions and, in turn, actual behavior (Ajzen, 1991). Ajzen (2011) highlighted the flexibility of the TPB, noting that it can integrate new predictors provided they meet specific criteria and are empirically supported. Building upon its flexibility, many scholars extended the theory by incorporating various factors (Niemiec et al., 2020) based on their research requirements and goals. Owing to the importance of the Theory of Planned Behavior, we employed and extended it by incorporating a cognitive factor, such as climate change concern, to explore its impact on individuals' intentions to perform three specific types of pro-environmental actions, including energy saving behavior, waste sorting, and single-use plastic behavior.

Yadav and Pathak (2017) highlighted that young consumers were more willing to purchase environmentally friendly products. In contrast, Chen and Tung (2014) found that individuals with a strong ecological conscience are more willing to select green hotels, even when it involves higher costs, considering their commitment to sustainable practices. The Theory of Planned Behavior (TPB), widely recognized for its application across multiple fields, has demonstrated strong explanatory power, particularly in the study of environmentally sustainable behaviors (Yuriev et al., 2020). Indeed, the Theory of Planned Behavior served as a key model in explaining diverse forms of pro-environmental behavior, actions intended to reduce ecological harm and support sustainability including recycling behaviors (Botetzagias et al., 2015), sustainable travel choices (Wang et al., 2024), adoption of green electricity (Li and Li, 2024), environmentally conscious food selection (Li et al., 2024) and participation in environmental activism (Carfora et al., 2017). Pinho (2025) reported that environmental identity, climate change perceptions, and direct experiences predicted conservation, sustainable transportation, and food-related behaviors, with climate anxiety mediating effects on wellbeing. On the other hand, in an experimental context, future-oriented imagination interventions were shown to influence pro-environmental intentions and donation behavior, indicating that cognitive framing can alter sustainability choices (Shaw et al., 2025). A meta-analysis of 26 studies further showed stronger correlations between behaviors within the same domain than across domains, suggesting an underlying general pro-environmental tendency that interventions could target (Ciocîrlan et al., 2025). An age cohort study of Dutch young adults highlighted diverse emotional needs and barriers to sustainable behavior, emphasizing the importance of multi-level support to foster engagement and wellbeing (Venhof and Jeronimus, 2026).

In the current study, pro-environmental behavior is examined through three specific dimensions: waste sorting, energy saving, and reducing single-use plastics. Waste sorting involves separating recyclables from general waste, which reduces landfill pressure and supports material reuse, contributing to resource conservation. Energy-saving behavior, such as turning off unused lights, using energy-efficient appliances, and minimizing consumption, plays a key role in lowering emissions and mitigating climate change. Reducing single-use plastic usage helps prevent long-term pollution, especially in marine ecosystems. By choosing reusable alternatives, individuals can protect wildlife and decrease plastic contamination in natural environments.

### 2.1 Hypotheses development

**Figure 1:**
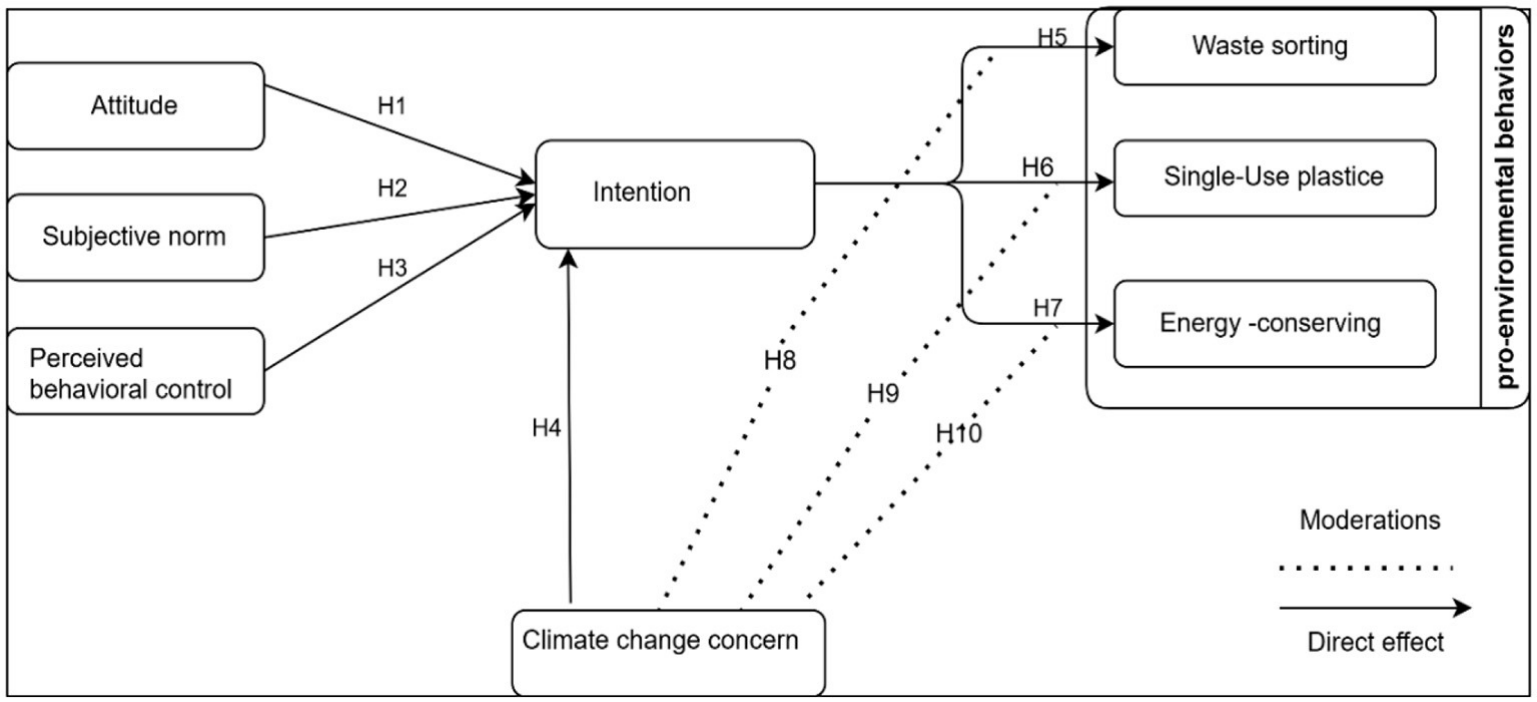
Pro-environmental behavior model.

Figure 1 outlines the conceptual model, which is based on the Theory of Planned Behavior (TPB). This theory suggests that an individual's actions are shaped by their attitude toward the behavior, the influence of perceived social pressure (subjective norms), and the extent to which they feel capable of carrying out the behavior (perceived behavioral control). Together, these psychological constructs offer insight into the formation of behavioral intentions, which are considered strong indicators of whether the behavior will be performed. In the context of pro-environmental behaviors, such as waste sorting, energy saving, and reducing single-use plastic. This study also incorporates climate change concern as a moderating variable, reflecting an individual's awareness of and concern for environmental issues.

**Behavioral attitude**, as conceptualized within TPB, refers to an individual's psychological tendency to evaluate a specific behavior as favorable or unfavorable, which influences the likelihood of performing that behavior (Ajzen, 1991). In the sustainability domain, previous research demonstrated that attitudes predict behavioral intention for different kinds of pro-environmental behaviors (Laureti and Benedetti, 2018; Kumar et al., 2020). In the present study, it is anticipated that individuals with favorable attitudes toward climate change will exhibit stronger intentions to engage in pro-environmental actions, including energy saving, waste sorting, and reducing the use of single-use plastics. Based on the literature, the current study proposes the following hypothesis:

**H1:** There is a positive impact of attitude toward climate change intention.

**Subjective norms (SNs)** refer to the perceived social pressure from important individuals, such as family members and friends, to perform or avoid certain behaviors (Ajzen, 1991; Han, 2015). When individuals believe that engaging in specific actions, such as pro-environmental behaviors, is supported or expected by their social circle, they are more likely to adopt those behaviors (Wu et al., 2019). In the context of sustainability, subjective norms play a significant role in influencing intentions for various environmental behavior (Li et al., 2018). However, existing literature presents mixed findings regarding the influence of subjective norms in different context while many studies confirm a positive relationship, others have found no significant impact on intention (Paul et al., 2016). These inconsistencies suggest the need for further investigation to clarify the impact of subjective norms in different environmental contexts. In the current research, subjective norms are evaluated by considering the extent to which individuals' intentions are shaped by the opinions and expectations of friends, family, community leaders, and governmental bodies. Accordingly, the following hypothesis is formulated:

**H2:** Subjective norms related to climate change positively influence the intention to engage in pro-environmental behaviors.

**Perceived behavioral control** (PBC) is defined as an individual's belief in their ability to successfully execute a specific behavior, considering the ease or difficulty of the task. It involves assessing various factors such as time, energy, prior experience, and other constraints that may affect one's ability to perform the behavior (Ru et al., 2018). When individuals perceive fewer barriers to action, their intention to engage in a particular behavior tends to strengthen (Hu et al., 2021). In the context of sustainability, Research consistently underscores the importance of PBC in predicting both intentions and actual behaviors (Ruangkanjanases et al., 2020). Therefore, based on this theoretical framework, the present study proposes the following hypothesis:

**H3:** Perceived behavioral control is positively related to the intention to adopt pro-environmental behaviors.

**Climate change concern (CCC)** is defined as individuals' awareness and understanding of environmental issues (Shimbar, 2021). The impact of climate change concern on individual behavioral decisions has received considerable attention in past studies (Yuriev et al., 2020). Research confirms that enhancing residents' concern about climate change is key to fostering community engagement in pro-environmental behaviors (Hernández-Moreno and Alcántara-Ayala, 2017). Previous research consistently highlights that concern about climate change is positively associated with various types of pro-environmental intentions and behaviors (Talwar et al., 2021; Tam and Chan, 2017). Building on this foundation, we propose a link between climate concern and intentions related to three specific pro-environmental behaviors: waste sorting, single-use plastic reduction, and energy conservation. Individuals who express greater concern for the environment are more likely to form stronger intentions to act on climate change. Thus, the following hypotheses are proposed:

**H4**: Climate change concern is positively associated with the intention to adopt pro-environmental.

**Climate change intentions (ITCC)**, as outlined in the theory of planned Behavior (TPB), represent an individual's commitment to performing a specific behavior and serve as key predictors of actual actions (Ajzen, 1991). Within sustainability research, it is well-established that individuals' intentions to engage in pro-environmental behavior are reliable predictors of their actual actions (Han et al., 2022). Stronger intentions to engage in pro-environmental practices such as waste reduction or the use of sustainable products are likely to translate into corresponding behaviors (Zhang et al., 2020). In contexts like food waste management, intentions significantly mediate the relation between social norms and waste-reducing behaviors, emphasizing the role of motivation and purpose in driving sustainable practices (Graham-Rowe et al., 2015). Similarly, in industries such as construction and manufacturing, green behavioral intentions have been shown to influence practices like recycling and eco-friendly collaborations (Tommasetti et al., 2018). Thus, behavioral intentions serve as fundamental drivers of sustainable actions, effectively bridging the gap between motivation and tangible outcomes across diverse contexts. Based on these insights, the following hypotheses are proposed to further explore the intention-behavior relationship in the domain of climate change:

**H5:** Climate change intentions have a positive influence on individuals' waste sorting behavior.

**H6:** Climate change intentions have a positive influence on individuals' energy-saving behavior.

**H7:** Climate change intentions have a positive influence on individuals' reduction of single-use plastic behavior.

Climate change concern has been widely examined as a moderating variable in understanding its impact on pro-environmental behaviors, including waste management and resource conservation (Wang et al., 2020). As a moderator, climate change concern can influence the strength or direction of the relation between various psychological or behavioral constructs. For example, Das and Ramalingam (2022) found that individuals with higher levels of climate change concern assign greater value to environmentally responsible actions, thereby enhancing their commitment to sustainable behaviors. In line with previous findings, the present study investigates the moderating role of climate change concern in the relation between pro-environmental intentions and actual behaviors. Accordingly, the following hypotheses are proposed:

**H8:** Climate change concern moderates the relationship between intention and waste sorting behavior.

**H9:** Climate change concern moderates the relationship between intention and single-use plastic reduction behavior.

**H10:** Climate change concern moderates the relationship between intention and energy-saving behaviors.

## 3 Materials and methods

### 3.1 Survey design and data collection

To validate the proposed hypotheses, this study utilized data from two countries, China and Pakistan, which have some cultural and socioeconomic similarities and differences. By analyzing two separate datasets from each country, the study ensured reliability and strengthened the credibility of its findings. The data from China was collected from Hunan province, known for its rich cultural heritage and significant economic role within the country. Meanwhile, the data from Pakistan was collected from Punjab province, which is one of the country's most populous and economically diverse regions.

Data collection was done using an online self-administered questionnaire, a method well-established for gathering substantial quantitative data (Khan et al., 2024). The questionnaire was distributed through Google Forms in Pakistan and SurveyMonkey in China. Data collection conducted from 1st January 2024 to 6 June 6, 2024. To maximize reach, survey links were shared across various social media platforms, including Facebook, Twitter, university WeChat groups, and QQ groups. Participation was open to any user of these social networks who was at least 18 years old. The questionnaire was originally developed in English for Pakistani participants. On the other hand, they were translated into Chinese for Chinese participants using a standard translation and review process. To make sure the translation equivalence, bilingual experts review the translated version to make sure the conceptual consistency and language clarity. Minor wording adjustments were made where needed to keep the original meaning of the items. This procedure was intended to minimize the potential language bias and help to enhance the comparability of responses across both countries.

After participants agreed to the terms outlined in the consent form, which included the researchers' contact details and emphasized that participation was voluntary and completely anonymous, they were directed to the questionnaire survey page. The survey consists of three main sections: Section A collected demographic details of the participants, Section B focused on participants' intentions toward climate change, their attitudes, subjective norms, and perceived behavioral control regarding three different pro-environmental behaviors. In Section C, participants report their actual behaviors related to waste sorting, energy conservation, and single-use plastic reduction. Three hundred eighty respondents completed the survey correctly, with 195 from China and 185 from Pakistan.

### 3.2 Measures

To ensure the accuracy and consistency of the findings, this study employs measurement scales grounded in established research, with necessary adaptations to fit the specific context and objectives of our study. The questions related to the Theory of Planned Behavior (TPB) constructs were adapted from Masud et al. (2016). The constructs pertinent to TPB were evaluated with a four-item scale. For instance, one of the sample items in Intention toward climate change (ITC) was “I'll do everything that can reduce the impact of climate change.” Similarly, Attitudes toward climate change (ATCC) were measured with the statement, “Climate change damages the natural environment and wildlife in Pakistan/China.” In the same way, Subjective norms (SN) were assessed with a sample item being “My family or peers frequently discuss climate change or global warming.” Furthermore, Perceived Behavioral Control (PBC) was measured with items such as, “I'm able to contribute to reduce CO_2_ emission through adaptation.”

The moderating variable Climate change concern (CC) was examined using four items adapted from Aleixo et al. (2021) including, “I am concerned about the effects of climate change.” Lastly, the items used to measure the three pro-environmental behaviors (PEB): waste sorting, single-use plastic (SUP) reduction, and energy conservation behavior (ECB) were adopted from various established sources (Wang et al., 2020; Oludoye et al., 2024; Walton and Austin, 2011; López-Mosquera et al., 2015). Waste sorting behavior was assessed with three items, one of which is “How often do you sort kitchen waste in your daily life?”. Single-use plastic (SUP) reduction behavior was measured using five items, including “I refuse plastic shopping bags by using reusable alternatives.” Similarly, energy conservation behavior (ECB) was evaluated with six items, with one example being “I turn off all energy-consuming appliances when they are not in use.” Higher scores on these scales indicate a greater level of the specific constructs being assessed. All items were measured using 5-point Likert scales (1 = Strongly Disagree, 5 = Strongly Agree). Detailed item listings are provided in the Appendix.

Indeed, the questionnaire items were adopted from prior literature and have been previously used in both Pakistani and Chinese contexts, ensuring cultural relevance. For Pakistani participants, the English version of the questionnaire was administered directly, as it was already in English. For Chinese participants, the items were translated into Chinese following a procedure like He et al. (2022), where two bilingual Chinese doctoral students employed the translation committee approach (Van de Vijver and Leung, 1997). A pilot test was conducted with friends and other students to check clarity and comprehension, and several items were revised based on their feedback to enhance the understanding and ensure cross-language equivalence.

### 3.3 Data analysis

To analyze our dataset from 380 participants, we consider SPSS version 26 and SmartPLS 4.0. SPSS was employed for descriptive statistics to summarize demographic characteristics of our sample. Overall, it provides a brief overview of pro-environmental behaviors among participants. On the other hand, Smarts PLS was employed for testing the proposed theoretical model. Hair et al. (2021) report that two approaches for structural equation modeling (SEM) can be used: covariance-based SEM (CB-SEM) and Variance-based SEM (Partial Least Squares SEM or PLS-SEM). The choice between these approaches depends on the objective and nature of the study (Dash and Paul, 2021). We adopted the PLS-SEM approach as we tested a theoretical framework from a predictive perspective, examining complex path correlations among multiple independent variables at various levels (Sabol et al., 2023). Second, its capacity to accommodate smaller sample sizes (Hair et al., 2010) makes it the appropriate method for hypothesis testing, considering our sample was modest.

The data analysis using PLS-SEM followed a two-step process. In the first process, Confirmatory Factor Analysis (CFA) was conducted to assess the measurement model. Also, the instrument validity and reliability were examined using different metrics, including internal consistency (Cronbach's alpha and composite reliability), convergent validity (average variance extracted or AVE), and discriminant validity (Fornell and Larcker criterion, 1981). In the second process, the structural model was analyzed to evaluate all proposed research hypotheses. For instance, the analysis for testing hypotheses H1–H7 involved a bootstrapping procedure with 5,000 subsamples to determine the significance of the hypothesized relationships between Latent constructs. After that, the moderation analysis for hypotheses H8–H10 was conducted to examine the moderating effect of climate change concern. Finally, a multi-group analysis for hypothesis H11 was performed to compare differences between the participants in China and Pakistan.

## 4 Empirical results

### 4.1 Descriptive statistics

Data were collected from 380 valid respondents, including 185 from Pakistan and 195 from China as reported in Table 1. In Pakistan, 47.6% of participants were male and 52.4% were female, whereas in China, 63.1% were male and 36.9% were female. Most respondents were aged between 18 and 35 years (Pakistan: 79.5%; China: 77.0%). Regarding education, the majority held graduate or higher degrees, with master's degrees accounting for 36.2% in Pakistan and 45.1% in China, while doctoral degrees were more common in China (16.9%) than in Pakistan (4.9%). In terms of residence, approximately two-thirds of participants in both countries lived in urban areas (Pakistan: 64.3%; China: 64.6%), with the remaining respondents residing in rural areas.

**Table 1:** Demographic characteristics of respondents.

**Variables**	**Category**	**Complete sample**	**Pakistan**		**China**	
		**Frequency**	**Frequency**	**%**	**Frequency**	**%**
Gender	Male	211	88	47.6	123	63.1
	Female	169	97	52.4	72	36.9
Age	18–25	141	74	40	67	34.4
	26–35	156	73	39.5	83	42.6
	36–45	73	35	18.9	38	19.5
	46–65	10	3	1.6	7	3.6
Education	Intermediate	48	27	14.6	21	10.8
	Graduate	135	82	44.3	53	27.2
	Master	155	67	36.2	88	45.1
	PhD	42	9	4.9	33	16.9
Area	Rural	135	66	35.7	69	35.4
	Urban	245	119	64.3	126	64.6

### 4.2 Test for common method bias

Collecting data through a self-reported questionnaire, where the same data sources and survey context are used to measure multiple constructs, may lead to systematic errors, such as common method bias (CMB; Podsakoff et al., 2012). To mitigate the risk of common variance issues and enhance the reliability of the data, we apply both a procedural and a statistical approach. In the procedural approach, we obtained consent before the data collection and assured the respondents of their anonymity and confidentiality (Podsakoff et al., 2003). We emphasized that no “correct” or “incorrect” answers exist. However, their response should be honest. Furthermore, we employed the existing validated items to measure the constructs of the study (Podsakoff and Organ, 1986). For the statistical approach, we used two methods to control for response bias: Harman's one-factor test and the Variance Inflation Factor (VIF) test. Harman's one-factor test results indicate that the unrotated single factor accounted for only 20.83% of variance, well below the 50% threshold (Fuller et al., 2016). Multi-collinearity was assessed via VIF (Kock, 2015), where all VIF values were below the recommended threshold of 3.3, as reported in Table 2. Therefore, the data were free from collinearity issues; thus, the standard method bias was not a problem in the proposed model.

**Table 2:** Reliability and validity.

**Construct**	**CR**			**AVE**			**Alpha**			**VIF**		
	**CN**	**PK**	**Complete**	**CN**	**PK**	**Complete**	**CN**	**PK**	**Complete**	**CN**	**PK**	**Complete**
ATTC	0.925	0.93	0.93	0.8	0.796	0.781	0.894	0.915	0.907	1.07	1.2	1.074
CCC	0.931	0.94	0.93	0.8	0.806	0.789	0.901	0.92	0.911	1.25	1.2	1.205
ECB	0.936	0.93	0.93	0.7	0.703	0.713	0.923	0.915	0.92	1.08	1.2	1.107
ITCC	0.93	0.9	0.92	0.8	0.712	0.742	0.9	0.865	0.884	1.34	1.3	1.291
PBC	0.943	0.93	0.93	0.8	0.78	0.794	0.92	0.906	0.914	1.19	1.3	1.15
SN	0.881	0.86	0.88	0.7	0.621	0.65	0.828	0.815	0.822	1.09	1.1	1.079
SUP	0.914	0.92	0.92	0.7	0.699	0.704	0.897	0.891	0.894	1.03	1.3	1.116
WSB	0.901	0.91	0.9	0.8	0.772	0.765	0.848	0.856	0.852	1.14	1.2	1.168

ATTC, attitudes toward climate change; CCC; climate change concerns; ECB, energy saving behavior; ITCC, intention to adopt climate change; PBC, perceived behavioral controls; SN, subjective norms; SUP, single-use plastics reduction behaviors; WSB, waste sorting behavior; CR, composite reliability; AVE, average variance extracted; VIF, Variance inflation factor.

### 4.3 Reliability and validity test of the questionnaire

The reliability was evaluated by assessing internal consistency, individual indicator reliability, convergent validity, and discriminant validity (Hair et al., 2019, 2021). The scales' reliability was evaluated through Composite Reliability (CR) and Cronbach's Alpha (CA). Convergent validity was determined by examining the Average Variance Extracted (AVE) and the factor loadings of the items. In contrast, discriminant validity was evaluated using the Fornell–Larcker criterion and the Heterotrait–Monotrait (HTMT) ratio. Table 2 demonstrates that Cronbach's α for each variable exceeds 0.8 across all samples. The composite reliability (CR) values range from 0.88 to 0.95 in the Chinese sample, 0.87–0.94 in the Pakistani sample, and 0.88–0.94 in the whole sample, demonstrating the reliability of internal consistency as results meet the threshold value of ≥ 0.7 (Hair et al., 2021). Similarly, the average variance extracted (AVE) for each construct exceeds 0.6 across all samples, with a threshold of 0.5. As reported in Table 3, all factor loadings were above 0.7 and higher than the respective cross-loading values. Therefore, it showed that the model achieved convergent validity. For discriminant validity, as indicated in Table 4, the square root of AVE for each variable for all samples was greater than the squared correlation between variables. Furthermore, all values in the HTMT matrix were below 0.85, offering strong validity evidence for all constructs across the samples (Fornell and Larcker, 1981). To ensure the robustness of these findings across countries, discriminant validity was also assessed separately for the Chinese and Pakistani samples, and results are presented in the Appendix. The results show a similar pattern, confirming the distinctiveness of constructs within each country and supporting the validity of the overall sample results.

**Table 3:** Factor loading in CFA analysis.

**Items**	**Factor loading**		
	**China**	**Pakistan**	**Complete**
**Attitudes toward climate change**			
ATTC1	0.862	0.911	0.882
ATTC2	0.862	0.899	0.884
ATTC3	0.885	0.86	0.882
ATTC4	0.867	0.895	0.888
**Climate change concerns**			
CCC1	0.892	0.908	0.903
CCC2	0.87	0.896	0.884
CCC3	0.923	0.913	0.918
CCC4	0.826	0.873	0.845
**Energy saving behavior**			
ECB1	0.845	0.874	0.866
ECB2	0.892	0.899	0.896
ECB3	0.761	0.761	0.763
ECB4	0.885	0.873	0.883
ECB5	0.89	0.873	0.881
ECB6	0.777	0.736	0.766
**Intention to adopt climate change**			
ITCC1	0.849	0.828	0.834
ITCC2	0.91	0.838	0.879
ITCC3	0.871	0.865	0.867
ITCC4	0.878	0.844	0.863
**Perceived behavioral control**			
PBC1	0.936	0.905	0.923
PBC2	0.885	0.879	0.883
PBC3	0.884	0.866	0.876
PBC4	0.883	0.882	0.88
**Subjective norms**			
SN1	0.706	0.884	0.795
SN2	0.848	0.822	0.835
SN3	0.82	0.680	0.772
SN4	0.855	0.752	0.819
**Single use—plastics reduction behavior**			
SUP1	0.816	0.801	0.808
SUP2	0.873	0.841	0.856
SUP3	0.898	0.907	0.902
SUP4	0.851	0.901	0.886
SUP5	0.762	0.717	0.732
**Waste sorting behavior**			
WSB1	0.913	0.896	0.903
WSB2	0.865	0.851	0.86
WSB3	0.823	0.888	0.86

**Table 4:** Discriminant validity assessment.

**Construct**	**ATTC**	**CCC**	**ECB**	**ITTC**	**PBC**	**SN**	**SUP**	**WSB**
**Fornell–Larcker criterion**								
ATTC	0.884							
CCC	0.154	0.888						
ECB	0.183	0.231	0.844					
ITTC	0.183	0.236	0.199	0.861				
PBC	0.132	0.276	0.106	0.242	0.891			
SN	0.121	0.214	0.151	0.209	0.166	0.806		
SUP	0.18	0.226	0.114	0.157	0.200	0.147	0.839	
WSB	0.096	0.062	0.104	0.36	0.125	0.149	0.166	0.875
**Heterotrait–Monotrait (HTMT) ratio**								
ATTC								
CCC	0.169							
ECB	0.195	0.232						
ITTC	0.200	0.258	0.215					
PBC	0.138	0.301	0.106	0.264				
SN	0.140	0.240	0.167	0.239	0.185			
SUP	0.192	0.245	0.129	0.177	0.223	0.171		
WSB	0.103	0.112	0.105	0.391	0.122	0.164	0.180	

ATTC, attitudes toward climate change; CCC, climate change concerns; ECB, energy saving behavior; ITTC, intention to adopt climate change; PBC, perceived behavioral control; SN, subjective norms; SUP, single-use-plastics reduction behavior; WSB, waste sorting behavior.

### 4.4 Structural equation modeling (SEM)

This section reports the structural model assessment by using path coefficients (Hair et al., 2017), coefficients of determination (*R*^2^), and effect sizes (*f*^2^). Path analysis was performed by using the bootstrapping technique to ensure robustness. Hypotheses H1–H7 were tested through this path analysis, while moderation analysis was employed for Hypotheses H8 through H10. Additionally, Partial Least Squares Multi-Group Analysis (PLS-MGA) was utilized to examine the statistically significant differences in path coefficients between the hypothesized models for China and Pakistan, corresponding to Hypothesis 11. Before these analyses, the structural equation model was tested for multicollinearity, with no issues detected across any of the models.

### 4.5 Path analysis

**Figure 2:**
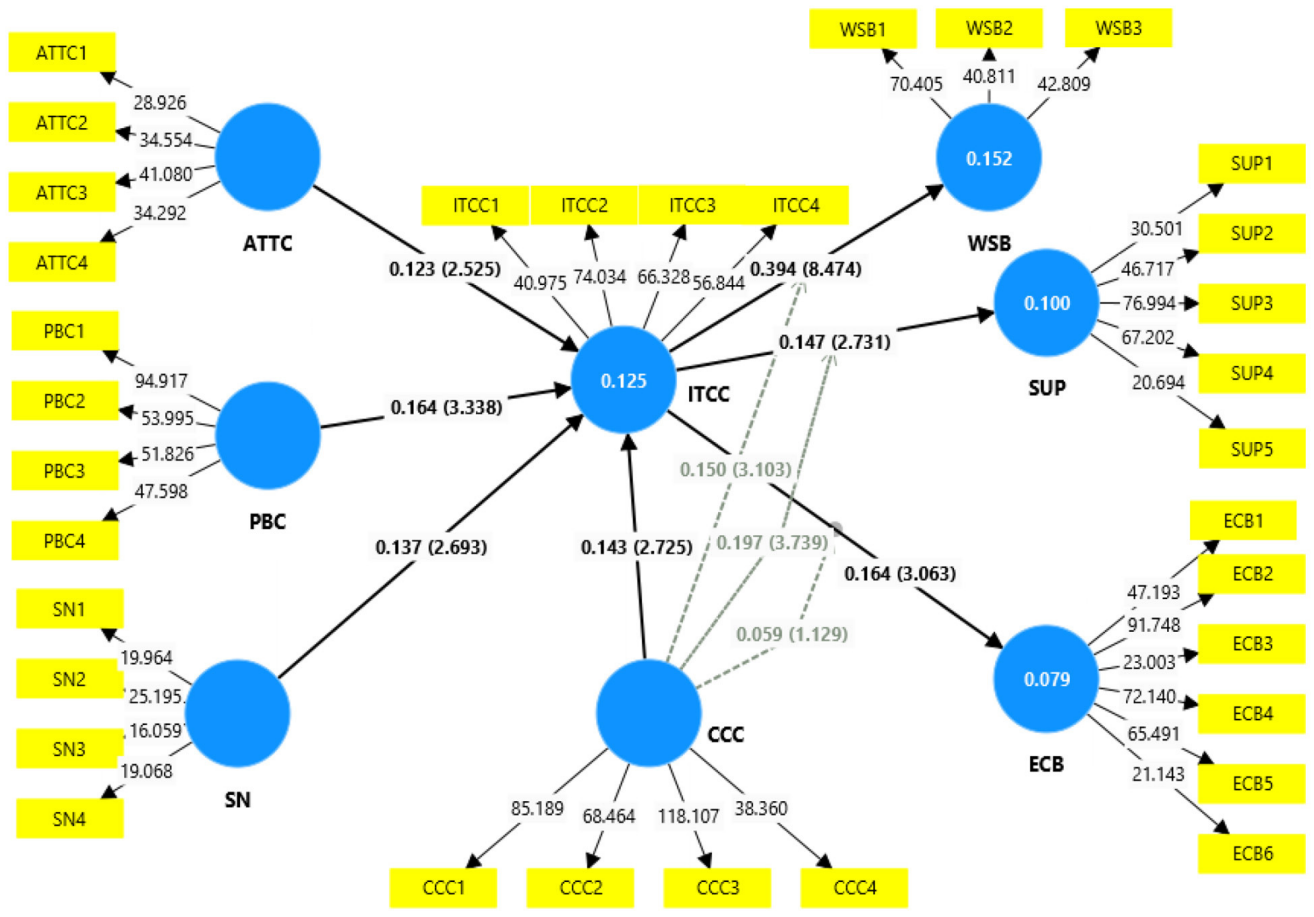
A path analysis of complete sample antecedents of the extended TPB model.

The significance of the proposed relation between the Theory of Planned Behavior (TPB) constructs and the intention to address climate change, as well as the moderating role of Climate Change Concern (CCC) on intention to climate change (ITCC) and pro-environmental behaviors (H1–H7), was tested using a bootstrapping analysis with 5,000 resamples. This analysis was performed for the entire sample as well as for each country separately. For the complete sample, the results of hypothesis testing are presented in Figure 2. The attitude (*p* < 0.01, *t* = 2.52), subjective norms (*p* < 0.007, *t* = 2.693), perceived behavioral control (*p* < 0.001, *t* = 3.338), and climate change concern (*p* < 0.006, *t* = 2.725) are all positively and significantly related to climate change intention. Furthermore, the climate change intention was found to be positively and significantly associated with waste sorting behavior (*p* < 0.000, *t* = 8.474), single-use plastic behaviors (*p* < 0.006, *t* = 2.731), and energy-saving behaviors (*p* < 0.002, *t* = 3.063). Overall, the results in Figure 2 support hypotheses H1–H8.

Overall results for the country-specific sample are generally consistent with those for the total sample and are shown in Table 5. However, Hypothesis H1: Attitude to Climate Change → Intention, (*p* < 0.9, *t* = 0.027) and Hypothesis H4 Climate Change Concern → Intention to Climate Change, (*p* < 0.2, *t* = 1.471) were not significant in the Pakistani sample, while they were significant in both the Chinese sample and the overall sample. Conversely, Hypothesis H6, Intention to Climate Change → Single-Use Plastic Reduction (*p* < 0.3, *t* = 0.919) was non-significant in the Chinese sample, but it was significant in the Pakistani sample and the overall sample.

**Table 5:** Results of hypothesis testing for Chinese and Pakistani participants.

**Parameter**	**China**				**Pakistan**			
**Direct effect**	β	***t***	***p***	**Decision**	β	***t***	***P***	**Decision**
ATTC -> ITCC	0.243^***^	3.482	0.001	Significant	0.013	0.172	0.800	Insignificant
SN -> ITCC	0.204^***^	2.843	0.004	Significant	0.166^***^	2.550	0.010	Significant
PBC -> ITCC	0.192^***^	3.105	0.002	Significant	0.247^***^	3.499	0.000	Significant
CCC-> ITCC	0.172^***^	2.442	0.010	Significant	0.113	1.471	0.140	Insignificant
ITCC -> ECB	0.186^**^	2.227	0.020	Significant	0.155^**^	1.922	0.050	Significant
ITCC -> SUP	0.093	0.925	0.355	Insignificant	0.201^***^	2.667	0.008	Significant
ITCC -> WSB	0.385^***^	5.444	0.000	Significant	0.386^***^	6.126	0.000	Significant

^***^and ^**^ indicate statistical significance at the 1% and 5%, respectively.

The explanatory power of the models was evaluated by using the coefficient of determination (*R*^2^), which indicates the proportion of variance in the dependent variable explained by the independent variables. According to Cohen (1988), *R*^2^ values are classified as significant (0.26), moderate (0.13), and weak (0.02). However, acceptable *R*^2^ values may vary depending on the research context. The results shows that the *R*^2^ values for the intention to combat climate change (ITCC) were 0.125 for the complete sample, 0.169 for the Chinese sample, and 0.120 for the Pakistani sample. In other words, the models explain 12.5%, 16.9%, and 12.0% of the variance in ITCC, respectively. For energy-saving behavior, the *R*^2^ values were 7.3% for the complete sample, 7.0% for the Chinese sample, and 12.7% for the Pakistani sample. The *R*^2^ values for single-use plastic reduction behavior were 10.0% for the complete sample, 5.0% for the Chinese sample, and 17.9% for the Pakistani sample. Finally, for waste sorting behavior, the *R*^2^ values were 15.2% for the complete sample, 13.1% for the Chinese sample, and 19.1% for the Pakistani sample.

### 4.6 Moderation analysis

**Figure 3:**
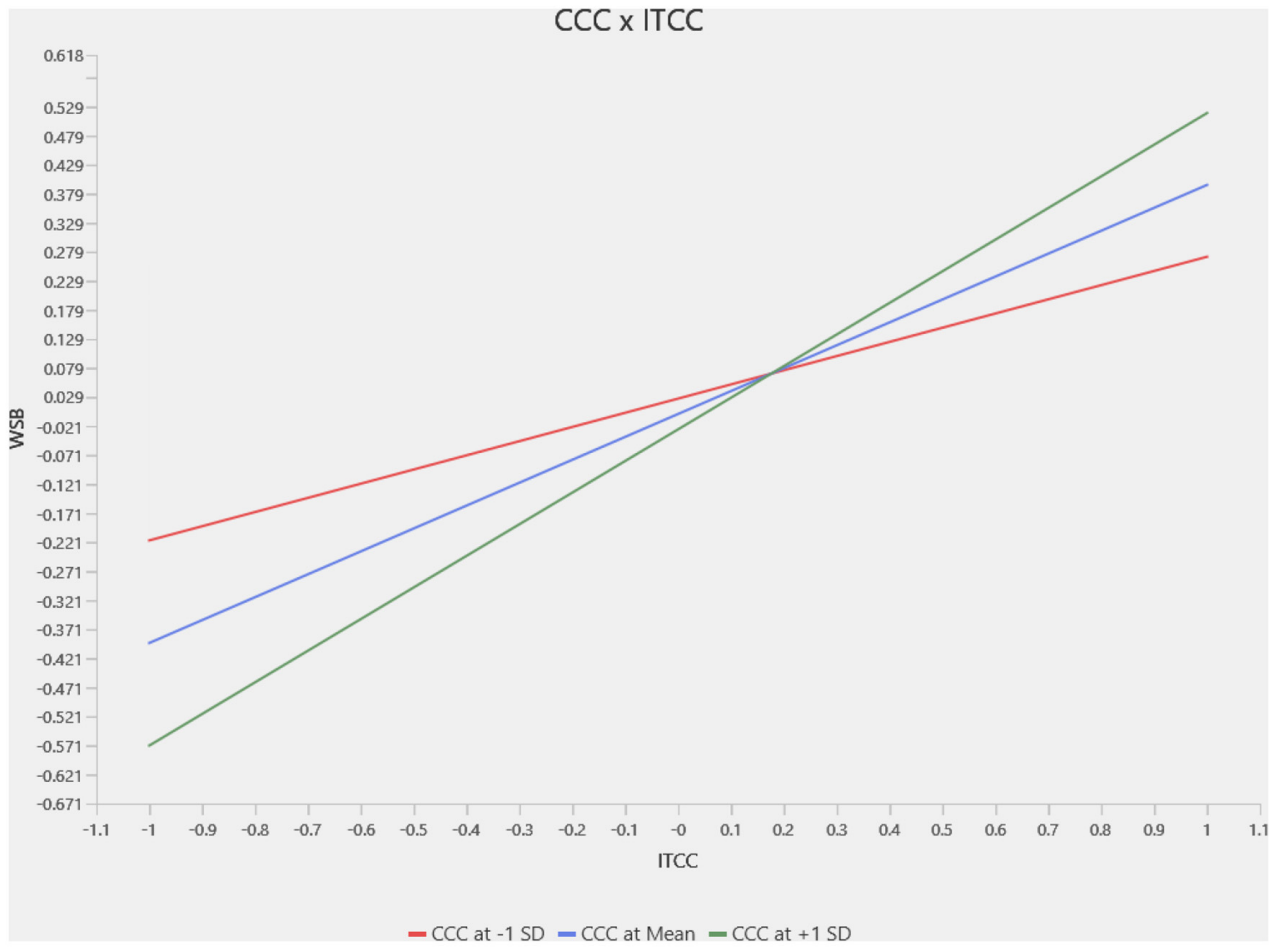
Moderator slope analysis—CCC × ITCC -> WSB.

**Figure 4:**
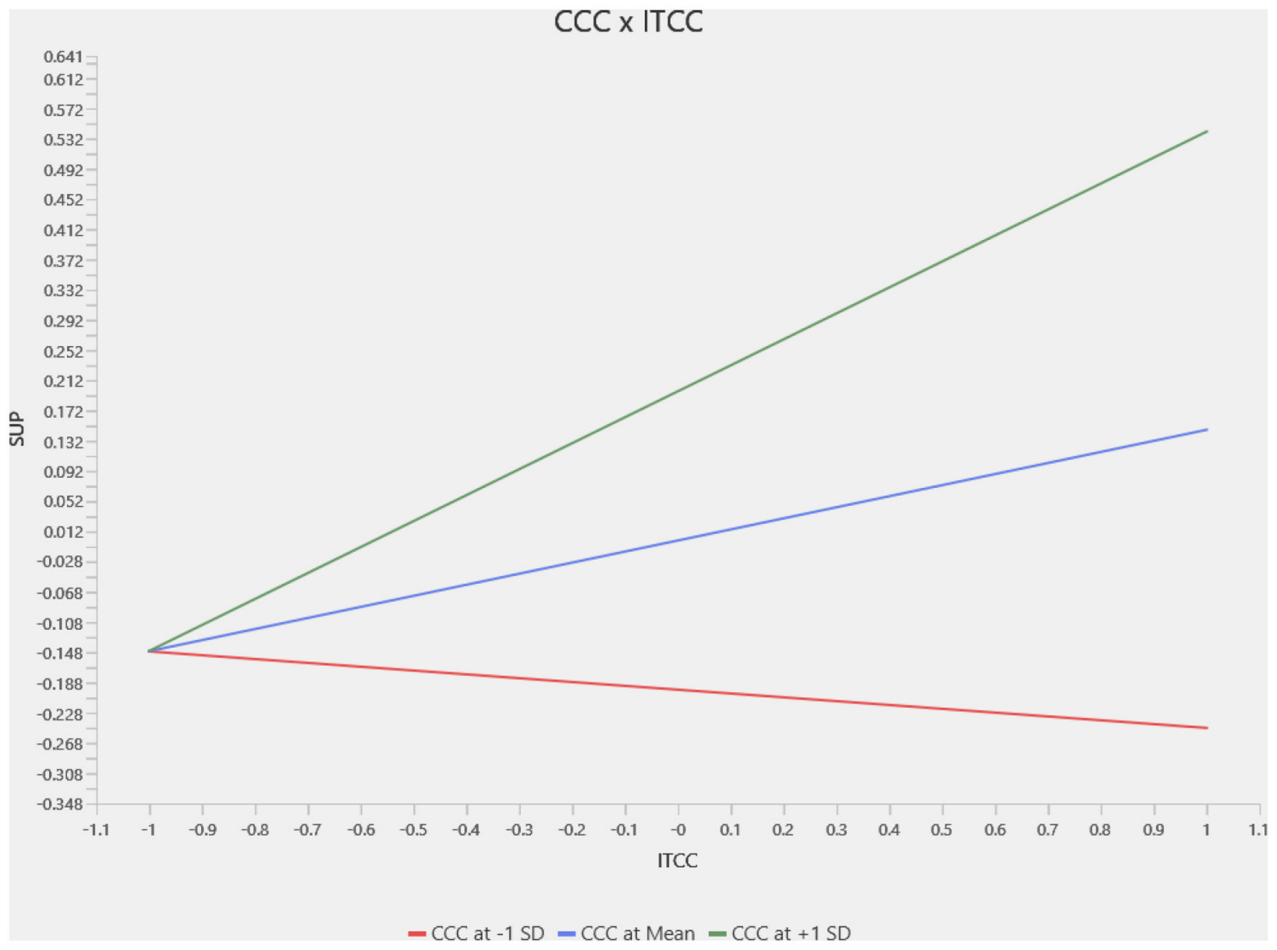
Moderator slope analysis—CCC × ITCC -> SUP.

**Figure 5:**
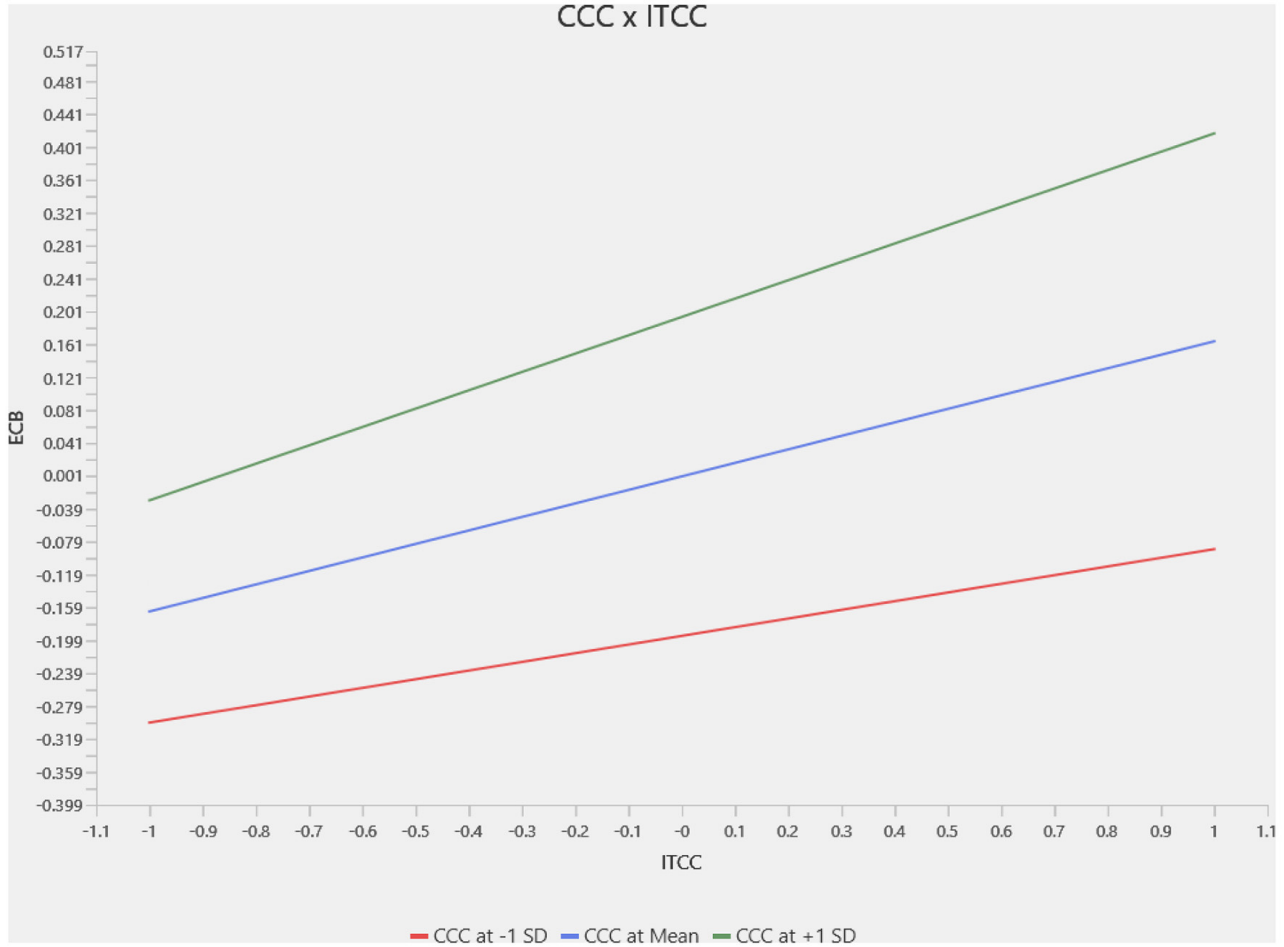
Moderator slope analysis—CCC × ITCC -> ECB.

This section presents a slope analysis to examine the moderating impact of Climate Change Concern (CCC) on the relationships between Intention to Climate Change (ITCC) and different pro-environmental behaviors. The results for the moderation analysis for the complete sample are in Figures 3–5, where we identified a positive and significant moderating effect of Climate Change Concern on the relation between Intention to Climate Change and Waste Sorting Behavior (WSB; *p* < 0.002, *t* = 3.103), as shown in Figure 3. Similarly, we identified that Climate Change Concern moderates the relation between Intention to Climate Change and Single-Use Plastic Reduction (SUP; *p* < 0.000, *t* = 3.739) as shown in Figure 4. Furthermore, climate change concern did not significantly moderate the relationship between intention to climate change and energy saving behavior, as shown in Figure 5.

The country-specific moderation analysis results are reported in Table 6. For the waste sorting behavior, climate change concern did not significantly moderate the relation between intention to take climate action and waste sorting behavior for the Chinese sample. In contrast, it significantly moderated for the Pakistani sample. For the single-use plastic reduction, climate change concern significantly moderated the relationship between intention and climate change concern and single-use plastic reduction, as the *p*-value is statistically significant for both samples. Additionally, we find that for the energy-saving behavior, climate change concern did not moderate the relationship between intention and climate change concern. Energy-saving behavior for both countries is shown in Table 6.

**Table 6:** Moderating effect of climate change intention.

**Parameter**	**China**			**Pakistan**		
	β	***t***	***p***	β	***t***	***p***
CCC × ITCC -> WSB	0.070	0.975	0.330	0.225^***^	3.514	0.001
CCC × ITCC -> SUP	0.167^**^	2.126	0.034	0.217^***^	2.951	0.003
CCC × ITCC -> ECB	0.117	1.595	0.111	−0.012	0.171	0.864

^***^and ^**^ indicate statistical significance at the 1% and 5%, respectively.

### 4.7 Multi-group analysis

The objective of Hypothesis H11 was to determine whether the relationships between the Theory of Planned Behavior (TPB) constructs, Intention to Combat Climate Change (ITCC), and pro-environmental behaviors differed between the two country samples. To investigate this, a Multigroup Analysis (MGA) was conducted to assess whether there were statistical differences between the Chinese and Pakistani participants. PLS-MGA was employed to compare the path coefficients across the two samples. The results revealed that the effect of Attitude toward Climate Change (ATTC) on ITCC was statistically significant. For instance, the influence of attitude on intention was stronger among Chinese participants compared to their Pakistani counterparts. However, the effects were not significant, but the trend of all other relationships was stronger among Pakistani respondents. Detailed results are presented in Table 7.

**Table 7:** Multigroup analysis.

**Effect**	**Difference (Chinese–Pakistan)**	***P***
ATTC -> ITCC	0.230	0.020^**^
CCC -> ECB	−0.152	0.158
CCC -> SUP	−0.153	0.136
CCC -> WSB	−0.014	0.899
ITCC -> ECB	0.031	0.781
ITCC -> SUP	−0.108	0.389
ITCC -> WSB	−0.001	0.999
PBC -> ITCC	−0.055	0.549
SN -> ITCC	0.037	0.694

^**^indicates the differences are significant in the relationships between the two countries at a 5% significance level.

## 5 Discussion on the findings

This section first summarizes the important findings and then compares them with the existing literature, along with the logical reasons to highlight their importance for policy suggestions. In this paper, we examined pro-environmental behavior based on the extended Theory of Planned Behavior by utilizing a dataset of 380 participants from China and Pakistan.

We mainly considered energy conservation, single-use plastic reduction, and waste sorting behavior simultaneously. Additionally, we investigated the results for the whole sample and China and Pakistan individually. The two countries, China and Pakistan, have some similarities which help us to make an analysis at aggregate level and also some differences in terms of economic development, environmental laws, governance ability, and infrastructure development from a national development perspective, which encourage us to carry out the analysis at the individual level. It provides meaningful contextual concepts to explain findings. China is a middle-income nation on the one hand, whereas Pakistan is a lower-middle-income country. On the second side, the economy of China has invested in environmental infrastructure that incorporates waste sorting systems, renewable energy development, and decent regulatory systems. Pakistan is experiencing institutional and infrastructural constraints, whereby the enforcement of environmental regulations cannot be enforced. Interestingly, the differences in the development level, in turn, can influence the translation of individual attitudes and intentions into pro-environmental behavior (Pontes et al., 2024). Thus, this contextual segregation is of significance to comprehend the recognized cross-country disparities in behaviors. Owing to these reasons, country-specific analysis was important in the study.

Our data passes through various tests to confirm the reliability before utilizing the Structural Equation Model. The Structural Equation Model results show that attitude toward climate change, concern, subjective norms, and perceived behavior control are positive and significantly related to climate change intention for China and Pakistan. We find that the intention to reduce climate change significantly positively impacted waste sorting, energy saving, and single-use plastic behavior.

Country-specific results were aligned to the whole sample, confirming the robustness of our findings, which is essential for policy. Our results reveal that the attitude toward climate Change → Intention, and climate change concern → intention to climate change were insignificant for Pakistan, whereas statistically significant for China. It is found that the intention to climate change → single plastic usage reduction was more important for Pakistan. Moderation analysis shows that climate change concern and waste sorting behavior did not significantly moderate the relationships between intention to take climate action and waste sorting behavior for China. In contrast, it was moderated considerably for Pakistan. We find that the single plastic use reduction and climate change concern moderate significantly in the relationship between intention to climate change concern and single plastic use reduction for China and Pakistan. Indeed, we found that there was a dominant moderating influence of climate change concern that was evident in Pakistan, which may be a compensatory behavior in which the infrastructural and institutional support has its limitations. It is noted that weak environmental regulations can be replaced to some extent by developing countries that are concerned about the environment to strengthen the intention-behavior relationship. Conversely, in China, there might be developed mechanisms of waste disposal and energy saving to reduce the peripheral effect of concern because the behavioral compliance is not as dependent on personal motivation. Our results highlight climate change concerns about energy saving, waste sorting behavior, and single-use plastic reduction.

Our results are aligned with those of (Nitu-Antonie and Feder 2017), who established the Theory of Planned Behavior for predictive pro-environmental behavior, suggesting that the model yields consistent results across countries and cultures. Further, our results reveal that including environmental concern as a moderating factor significantly enhances the model's explanatory power, particularly for single-use plastic reduction behaviors. It is consistent with the work of (Hidalgo-Crespo and Amaya-Rivas 2024), who found that including external influences as a moderator increased the model's predictive power for waste reduction behavior in America.

Furthermore, the path analyses revealed that each construct within the Theory of Planned Behavior (TPB) framework robustly predicts climate change intentions. Specifically, attitudes exerted a positive and significant effect on the intention to adopt climate change mitigation behavior across all three pro-environmental domains: energy conservation (Qalati et al., 2022); waste sorting (Zhang et al., 2023), and reduction of single-use plastics (Gu et al., 2023; Oludoye et al., 2024; Wang et al., 2024).

However, the results from the multigroup analysis show that the influence of attitude on intention was observed only in Chinese respondents, with no significant effect in the Pakistani sample. This outcome may be explained through two main perspectives: cultural differences and economic aspects. First, from a cross-cultural perspective, our results are consistent with prior research highlighting significant behavioral differences among individuals from different nationalities (Lamiño Jaramillo et al., 2023; Phuphisith et al., 2020). Second, economic aspects play a crucial role in shaping this relationship. Research indicates that in emerging economies, attitudes tend to be a stronger predictor of pro-environmental intentions (Pontes et al., 2024). In contrast, this effect is usually diminished in developing countries, as Munir et al. (2019) signaled.

Our study demonstrates that the subjective norms had a positive and significant impact on intentions related to climate change mitigation across all pro-environmental behaviors and among participants from China and Pakistan, consistent with Zhang et al. (2024); Hu et al. (2024). It has been noted that subjective norms play a crucial role in shaping behavioral intentions, thereby facilitating the adoption of pro-environmental behaviors (PEBs; Nie and Kou, 2021; Zhang et al., 2020) as the subjective norms offer guidance on what is considered appropriate or inappropriate, as well as beneficial or undesirable actions (Schreinemachers et al., 2023). Similarly, the perceived behavioral control significantly impacted intentions to engage in energy conservation, waste sorting, and single-use plastic reduction behaviors. It is consistent with existing work (Ajzen, 1991; Alzubaidi et al., 2021; Guo et al., 2022). These results reveal that perceived behavioral control operates differently across development contexts. In China, well-established environmental infrastructure and public services can reduce reliance on individual perceptions of control, as institutions support the facilitations of pro-environmental behaviors. On the other hand, Pakistan has limited infrastructure facilities to focus on personal actions and construction of perceived behavioral control and intention as more decisive predictors of actual behavior. Indeed, in resource-constrained settings, individual motives play a stronger role in shaping environmental action.

These results align with theoretical expectations—all three factors were statistically significant and positively influenced intentions, though their coefficients varied in strength. The perceived behavioral control emerged as the strongest predictor of intentions to engage in pro-environmental behaviors, surpassing the effects of attitude and subjective norms, which were significant but had smaller coefficients. Regarding climate change concerns, the results indicate a positive correlation with climate change intentions, which is supported by existing studies that report a significant and positive impact of environmental concern on sustainable intentions (Maduku, 2024; Saari et al., 2021). However, country-specific results reveal that the effect of environmental concern on intentions was significantly observed only among Chinese participants.

Ajzen (2002) reported that an individual's intentions directly predict actual behavior, a crucial intermediary linking attitudes, subjective norms, and perceived behavioral control (PBC) to the resulting behavior. Our findings highlighted a strong impact of climate change-related intentions on actual behaviors, such as waste sorting, energy conservation, and the reduction of single-use plastics. It is consistent with prior research, demonstrating intentions' predictive power on behaviors in these contexts (Juma-Michilena et al., 2024; Oludoye et al., 2024). We found that reducing single-use plastics did not significantly predict behavior among Chinese participants. At the same time, it was significant and positive in the Pakistani sample, which suggests the need for enhanced efforts to promote single-use plastic reduction for the whole sample. Although some initiatives are underway, they are relatively limited compared to those in major cities in China (Sun and He, 2023) and Pakistan. Our moderation analysis shows that the climate change concern significantly moderates the relation between climate change intention and plastic reduction behavior. Specifically, high levels of climate change concern strengthen the relationship between climate change intention and the reduction of single-use plastics. It is aligned with Saari et al. (2021), who have shown that individuals with higher environmental concern are more likely to form intentions to engage in pro-environmental practices. Additionally, similar moderating effects of climate change concern have been observed in the context of waste sorting behavior (Hidalgo-Crespo and Amaya-Rivas, 2024).

To summarize, overall, our results confirm the explanatory strength of the extended Theory of Planned Behavior across the full sample and individually in the Chinese and Pakistani context. Attitude toward climate change, subjective norms, perceived behavioral control, and climate change concern significantly predicted intention to address climate change in the overall model, while intention consistently translated into energy-saving, waste sorting, and single-use plastic reduction behaviors. Moderation analysis further showed that climate change concern strengthened the intention-behavior relationship for waste sorting and single-use plastic reduction, but not for energy-saving behavior. Country-specific analyses revealed meaningful contextual differences: attitudes and climate change concern were significant predictors of intention only in the Chinese sample, whereas intention more strongly predicted single-use plastic reduction in the Pakistani sample. These differences likely reflect variations in infrastructure, regulatory enforcement, and economic constraints, whereas more developed environmental systems in China reduce reliance on individual intentions, while behavioral outcomes in Pakistan depend more heavily on personal motivation due to limited institutional support.

## 6 Conclusion and policy suggestions

This study examined the factors influencing pro-environmental behaviors in Pakistani and Chinese samples through the extended Theory of Planned Behavior (TPB) model, demonstrating its robust applicability across the countries. Our study simultaneously considered three main pro-environments, namely, energy conservation, single-use plastic reduction, and waste sorting, in Pakistan and China. Additionally, this paper finds Chinese and Pakistani groups as a sample for analysis, and each country's analysis is carried out separately.

Our results show that the attitude toward climate change, subjective norms, perceived behavioral control, and climate change concern are all positively and significantly related to climate change intention for the complete samples. Furthermore, intention to climate change was positive and significantly associated with waste sorting behavior, single-use plastic behavior, and energy-saving behavior. Additionally, we considered country-specific analyses, and the results are aligned with the full sample. Moreover, our results highlight that the Attitude to Climate Change → Intention and Climate Change Concern → Intention to Climate Change were insignificant for the Pakistani sample, whereas they were significant in the Chinese sample, whereas on the other hand, we find that the Intention to Climate Change → Single-Use Plastic Reduction was more significant in Pakistani sample. Our country-specific moderation analysis reveals that waste sorting behavior and climate change concern did not significantly moderate the relation between intention to take climate action and waste sorting behavior for China. In contrast, it significantly moderated in the case of Pakistan. Results further report that for single-use plastic reduction, climate change concern significantly moderated the relationship between intention to climate change concern and single-use plastic reduction for both countries. Overall, our findings emphasize the importance of climate change, particularly with the focus on energy saving, single-use plastic usage, and waste sorting behavior adjustment.

### 6.1 Theoretical Implications

The study makes two theoretical contributions by extending the Theory of Planned Behavior to provide a more comprehensive understanding of pro-environmental behaviors. First, it enhances the TPB framework by incorporating three distinct pro-environmental behaviors (energy saving, waste sorting, and single-use plastic use), allowing for an integrated analysis on how various underlying constructs influence multiple behaviors. This approach offers a broader perspective on the factors driving sustainable actions and urges for more exploration of behavioral patterns. Second, the study introduces climate change concern as a moderating variable between the intention to engage in climate-conscious behaviors and actual pro-environmental actions. Doing so highlights the importance of concern for climate change in strengthening the relationship between individuals' intentions and their corresponding behaviors. It adds depth to the TPB by demonstrating how specific concerns about environmental issues can impact the effectiveness of intention in predicting behavior.

Furthermore, the paper contributes to the literature by demonstrating behavioral heterogeneity across pro-environmental domains and national contexts. The differential strength of TPB paths across China and Pakistan suggests that TPB relations are context-sensitive rather than universally stable, supporting recent arguments for culturally contingent behavioral models. By revealing that certain TPB components (e.g., attitudes and environmental concern) function differently across countries, the study advances theoretical understanding by highlighting the need to integrate contextual and socio-economic conditions in behavioral theories of sustainability. This encourages future theoretical refinement of TPB-based models by incorporating cultural and structural boundary conditions.

### 6.2 Practical implications

The practical implications of this study enrich existing knowledge in several ways. First, the findings suggest that a comprehensive approach to policy development is crucial for effectively addressing global warming. By simultaneously evaluating a range of pro-environmental behaviors, policymakers and researchers can identify critical gaps between current practices and necessary actions, allowing for the creation of more targeted and impactful policies. Second, the study underscores the importance of localized strategies. Instead of adopting a one-size-fits-all approach, environmental policies should be tailored to different regions' specific challenges. By considering the unique environmental conditions of each province, these localized strategies can more effectively address local needs and improve policy outcomes. Furthermore, the study highlights the significance of culturally informed policy and community engagement. It reveals the strategies to be adopted to different cultural groups' social norms, attitudes, and competencies. For instance, in China, enhancing attitudes toward sustainability and fostering cultural competence could yield better results, while focusing on perceived behavioral control and subjective norms may be more effective in strengthening intentions to engage in climate-conscious behaviors in Pakistan. Moreover, promoting environmental concern through education and community initiatives can enhance the effectiveness of these strategies by indirectly influencing behaviors.

Moreover, the findings suggest that behavior-specific interventions are likely to be more effective than general environmental campaigns. Policymakers should design targeted initiatives that align with the dominant predictors of each behavior, for example, enhancing behavioral control mechanisms (e.g., infrastructure, accessibility, incentives) for waste sorting and plastic reduction, while strengthening normative messaging for energy conservation. Furthermore, integrating climate change education into formal curricula and public awareness programs can elevate environmental concern, thereby reinforcing the translation of intentions into actual behavior. Such evidence-based, behavior-tailored policies can improve policy efficiency and support long-term sustainability goals in both developing and emerging economies.

## 7 Limitations and future research

Like other studies, our work is not free from limitations. First, it employs a cross-sectional design, capturing attitudes and behaviors at a single point in time, whereas sustainable behaviors often require long-term commitment. Second, data were collected via online surveys, which may exclude older adults and/or the rural population. Third, pro-environmental behaviors were self-reported, potentially differing from actual actions. Fourth, the samples from China and Pakistan may be relatively small and not fully representative, which limits generalizability. Fourth, identical antecedent items were used across different pro-environmental behaviors, which may limit the ability to detect behavior-specific effects. Future research could address these limitations by adopting longitudinal designs to capture behavior over time, employing mixed methods such as interviews and direct observation to improve measurement accuracy, expanding the sample to include more diverse populations and regions, and examining additional factors that influence pro-environmental behavior to provide a more comprehensive understanding of sustainable actions. Additionally, measuring behavioral intention was at the general level and not behavior-specific, although the analysis was done on particular pro-environmental behaviors. Extant studies of TBS have indicated that high specificity with intention and behavior can be more predictive. In this way, behavioral-specific variation could be minimized when general intention measures are used. The future study should take into consideration the behavior-specific intention items to facilitate the intention-behavior congruency.

## Data Availability

The raw data supporting the conclusions of this article will be made available by the authors, without undue reservation.

## Appendix

**Appendix A1:** Discriminate validity assessment (Chinese sample).

ATTC	0.869							
CCC	0.134	0.879						
ECB	0.080	0.174	0.847					
ITCC	0.227	0.290	0.192	0.877				
PBC	-0.032	0.314	0.050	0.211	0.898			
SN	-0.009	0.337	0.208	0.228	0.168	0.808		
SUP	0.104	0.143	0.071	0.102	0.053	0.148	0.831	
WSB	0.083	0.086	0.135	0.358	0.039	0.110	0.065	0.868
**Heterotrait-monotrait ratio (HTMT)—matrix**								
ATTC								
CCC	0.148							
ECB	0.079	0.184						
ITCC	0.246	0.320	0.189					
PBC	0.062	0.340	0.060	0.224				
SN	0.090	0.349	0.233	0.248	0.167			
SUP	0.118	0.162	0.088	0.089	0.060	0.139		
WSB	0.107	0.125	0.128	0.369	0.073	0.126	0.058	

**Appendix A2:** Discriminate validity assessment (Pakistani sample).

**Fornell-Larcker criterion**								
**Construct**	**ATTC**	**CCC**	**ECB**	**ITCC**	**PBC**	**SN**	**SUP**	**WSB**
ATTC	0.892							
CCC	0.224	0.898						
ECB	0.256	0.306	0.837					
ITCC	0.128	0.180	0.229	0.846				
PBC	0.306	0.229	0.175	0.284	0.883			
SN	0.265	0.116	0.112	0.215	0.194	0.792		
SUP	0.236	0.304	0.171	0.246	0.367	0.199	0.836	
WSB	0.116	0.036	0.087	0.371	0.228	0.192	0.275	0.878
**Heterotrait-monotrait ratio (HTMT)—matrix**								
ATTC								
CCC	0.229							
ECB	0.284	0.335						
ITCC	0.145	0.194	0.241					
PBC	0.338	0.248	0.193	0.319				
SN	0.309	0.123	0.117	0.221	0.216			
SUP	0.262	0.332	0.194	0.275	0.406	0.213		
WSB	0.128	0.105	0.116	0.412	0.247	0.236	0.303	

## References

[B1] Ajzen I. (1991). The theory of planned behavior. Organ. Behav. Hum. Decis. Process. 50, 179–211. doi: 10.1016/0749-5978(91)90020-T

[B2] Ajzen I. (2002). Perceived behavioral control, self-efficacy, locus of control, and the theory of planned behavior 1. J. Appl. Soc. Psychol. 32, 665–683. doi: 10.1111/j.1559-1816.2002.tb00236.x

[B3] Ajzen I. (2011). The theory of planned behaviour: reactions and reflections. Psychol. Health 26, 1113–1127. doi: 10.1080/08870446.2011.613995 21929476

[B4] Aleixo A. M. Leal S. Azeiteiro U. M. (2021). Higher education students' perceptions of sustainable development in Portugal. J. Clean. Prod. 327:129429. doi: 10.1016/j.jclepro.2021.129429

[B5] Alzubaidi H. Slade E. L. Dwivedi Y. K. (2021). Examining antecedents of consumers' pro-environmental behaviours: TPB extended with materialism and innovativeness. J. Bus. Res. 122, 685–699. doi: 10.1016/j.jbusres.2020.01.017

[B6] Batool N. Wani M. D. Shah S. A. Dada Z. A. (2024). Theory of planned behavior and value-belief norm theory as antecedents of pro-environmental behaviour: evidence from the local community. J. Hum. Behav. Soc. Environ. 34, 693–709. doi: 10.1080/10911359.2023.2205912

[B7] Botetzagias I. Dima A. F. Malesios C. (2015). Extending the theory of planned behavior in the context of recycling: the role of moral norms and of demographic predictors. Resourc. Conserv. Recycl. 95, 58–67. doi: 10.1016/j.resconrec.2014.12.004

[B8] Carfora V. Caso D. Sparks P. Conner M. (2017). Moderating effects of pro-environmental self-identity on pro-environmental intentions and behaviour: a multi-behaviour study. J. Environ. Psychol. 53, 92–99. doi: 10.1016/j.jenvp.2017.07.001

[B9] Chen M. F. Tung P. J. (2014). Developing an extended theory of planned behavior model to predict consumers' intention to visit green hotels. Int. J. Hospital. Manage. 36, 221–230. doi: 10.1016/j.ijhm.2013.09.006

[B10] Ciocîrlan A. B. Baird H. Rowe R. (2025). A systematic review and meta-analysis of the relationships between pro-environmental behaviours. J. Environ. Psychol. 108:102807. doi: 10.1016/j.jenvp.2025.102807

[B11] Cohen J. (1988). Statistical Power Analysis for the Behavioral Sciences, 2nd Edn. Hillsdale, NJ: Lawrence Erlbaum Associates, Publishers.

[B12] Das M. Ramalingam M. (2022). What drives product involvement and satisfaction with OFDs amid COVID-19? J. Retail. Consum. Serv. 68:103063. doi: 10.1016/j.jretconser.2022.103063

[B13] Dash G. Paul J. (2021). CB-SEM vs PLS-SEM methods for research in social sciences and technology forecasting. Technol. Forecast. Soc. Change 173:121092. doi: 10.1016/j.techfore.2021.121092

[B14] Dietz T. Gardner G. T. Gilligan J. Stern P. C. Vandenbergh M. P. (2009). Household actions can provide a behavioral wedge to rapidly reduce US carbon emissions. Proc. Nat. Acad. Sci. U.S.A. 106, 18452–18456. doi: 10.1073/pnas.0908738106 19858494 PMC2767367

[B15] Faghani A. Valizadeh N. Bijani M. Fallah Haghighi N. (2023). Towards participation in pro-environmental activities: application of dual-pathway model of collective action. J. Agric. Sci. Technol. 25, 565–579. doi: 10.22034/jast.25.3.565

[B16] Fornell C. Larcker D. F. (1981). Evaluating structural equation models with unobservable variables and measurement error. J. Market. Res. 18, 39–50. doi: 10.1177/002224378101800104

[B17] Fu L. Zhang Y. Xiong X. Bai Y. (2017). Pro-environmental awareness and behaviors on campus: evidence from Tianjin, China. Euras. J. Math. Sci. Technol. Educ. 14, 427–445. doi: 10.12973/ejmste/77953

[B18] Fuller C. M. Simmering M. J. Atinc G. Atinc Y. Babin B. J. (2016). Common methods variance detection in business research. J. Bus. Res. 69, 3192–3198. doi: 10.1016/j.jbusres.2015.12.008

[B19] Graham-Rowe E. Jessop D. C. Sparks P. (2015). Predicting household food waste reduction using an extended theory of planned behaviour. Resour. Conserv. Recycl. 101, 194–202. doi: 10.1016/j.resconrec.2015.05.020

[B20] Gu F. Zhu Z. Ali S. (2023). Analysis of factors of single-use plastic avoidance behavior for environmental sustainability in China. Processes 11:1412. doi: 10.3390/pr11051412

[B21] Gulliver R. Wibisono S. Fielding K. S. Louis W. R. (2021). The Psychology of Effective Activism. Elements in Applied Social Psychology. Cambridge: Cambridge University Press. doi: 10.1017/9781108975476

[B22] Guo D. Wang X. Feng T. Han S. (2022). Factors influencing the waste separation behaviors of urban residents in Shaanxi Province during the 14th National Games of China. Int. J. Environ. Res. Public Health 19:4191. doi: 10.3390/ijerph19074191 35409874 PMC8998419

[B23] Hair J. Hollingsworth C. L. Randolph A. B. Chong A. Y. L. (2017). An updated and expanded assessment of PLS-SEM in information systems research. Indus. Manage. Data Syst. 117, 442–458. doi: 10.1108/IMDS-04-2016-0130

[B24] Hair J. F. Black W. C. Babin B. J. Anderson R. E. (2010). Multivariate Data Analysis, 7th Edn. Upper Saddle River, NJ: Prentice Hall.

[B25] Hair J. F. Risher J. J. Sarstedt M. Ringle C. M. (2019). When to use and how to report the results of PLS-SEM. Euro. Business Rev. 31, 2–24. doi: 10.1108/EBR-11-2018-0203

[B26] Hair J. F. Jr. Hult G. T. M. Ringle C. M. Sarstedt M. Danks N. P. Ray S. Ray S. (2021). “An introduction to structural equation modeling,” in Partial Least Squares Structural Equation Modeling (PLS-SEM) Using R. Classroom Companion: Business (Cham: Springer International Publishing), 1–29. doi: 10.1007/978-3-030-80519-7_1

[B27] Han H. (2015). Travelers' pro-environmental behavior in a green lodging context: converging value-belief-norm theory and the theory of planned behavior. Tourism Manage. 47, 164–177. doi: 10.1016/j.tourman.2014.09.014

[B28] Han M. S. Hampson D. P. Wang Y. Wang H. (2022). Consumer confidence and green purchase intention: an application of the stimulus-organism-response model. J. Retail. Consum. Serv. 68:103061. doi: 10.1016/j.jretconser.2022.103061

[B29] He R. Jin J. Qiu X. Zhang C. Yan J. (2023). Rural residents' climate change perceptions, personal experiences, and purchase intention–behavior gap in energy-saving refrigeration appliances in Southwest China. Environ. Impact Assess. Rev. 98:106967. doi: 10.1016/j.eiar.2022.106967

[B30] He Z. Liu Y. Liu X. Wang F. Zhu H. (2022). Influence of multi-dimensional environmental knowledge on residents' waste sorting intention: moderating effect of environmental concern. Front. Psychol. 13:957683. doi: 10.3389/fpsyg.2022.957683 36524171 PMC9745202

[B31] Hernández-Moreno G. Alcántara-Ayala I. (2017). Landslide risk perception in Mexico: a research gate into public awareness and knowledge. Landslides 14, 351–371. doi: 10.1007/s10346-016-0683-9

[B32] Hidalgo-Crespo J. Amaya-Rivas J. L. (2024). Citizens' pro-environmental behaviors for waste reduction using an extended theory of planned behavior in Guayas province. Clean. Eng. Technol. 21:100765. doi: 10.1016/j.clet.2024.100765

[B33] Hu B. Guo H. Zhou P. Shi Z. L. (2021). Characteristics of SARS-CoV-2 and COVID-19. Nat. Rev. Microbiol. 19, 141–154. doi: 10.1038/s41579-020-00459-7 33024307 PMC7537588

[B34] Hu X. Li M. Gu M. Zhang B. (2024). How online pro-environmental games affect users' pro-environmental behavioural intentions?—Insights from Ant Forest. J. Environ. Manage. 368:122182. doi: 10.1016/j.jenvman.2024.122182 39133965

[B35] Juma-Michilena I.-J. Ruiz-Molina M.-E. Gil-Saura I. Belda-Miquel S. (2024). Pro-environmental behaviours of generation Z: a cross-cultural approach. Int. Rev. Public Nonprofit Market. doi: 10.1007/s12208-024-00395-9

[B36] Kaya Kanli N. Küçükefe B. (2023). Is the environmental Kuznets curve hypothesis valid? A global analysis for carbon dioxide emissions. Environ. Dev. Sustain. 25, 2339–2367. doi: 10.1007/s10668-022-02138-4

[B37] Khan A. N. Mehmood K. Kwan H. K. (2024). Green knowledge management: a key driver of green technology innovation and sustainable performance in construction organizations. J. Innov. Knowl. 9:100455. doi: 10.1016/j.jik.2023.100455

[B38] Kirikkaleli D. Sofuoglu E. Abbasi K. R. Addai K. (2023). Economic complexity and environmental sustainability in the eastern European economy: evidence from a novel Fourier approach. Regional Sustain. 4, 349–358. doi: 10.1016/j.regsus.2023.08.003

[B39] Kock N. (2015). Common method bias in PLS-SEM: a full collinearity assessment approach. Int. J. e-Collab. 11, 1–10. doi: 10.4018/ijec.2015100101

[B40] Kumar S. Maurya V. K. Prasad A. K. Bhatt M. L. B. Saxena S. K. (2020). Structural, glycosylation and antigenic variation between 2019 novel coronavirus (2019-nCoV) and SARS coronavirus (SARS-CoV). Virus Dis. 31, 13–21. doi: 10.1007/s13337-020-00571-5 32206694 PMC7085496

[B41] Lamiño Jaramillo P. Tábora-Sarmiento S. Millares-Forno C. Boren-Alpízar A. E. (2023). The theory of reasoned action as a predictor of environmental behavior: a cross-cultural comparison between college students from Texas, Louisiana, and Honduras. J. Hum. Behav. Soc. Environ. 33, 521–536. doi: 10.1080/10911359.2022.2072040

[B42] Laureti T. Benedetti I. (2018). Exploring pro-environmental food purchasing behaviour: an empirical analysis of Italian consumers. J. Clean. Prod. 172, 3367–3378. doi: 10.1016/j.jclepro.2017.11.086

[B43] Li H. Cao A. Chen S. Guo L. (2024). How does risk perception of the COVID-19 pandemic affect the consumption behavior of green food? Environ. Dev. Sustain. 26, 2307–2329. doi: 10.1007/s10668-022-02819-0 36530362 PMC9734953

[B44] Li J. Li A. (2024). Optimizing electric vehicle integration with vehicle-to-grid technology: the influence of price difference and battery costs on adoption, profits, and green energy utilization. Sustainability 16:1118. doi: 10.3390/su16031118

[B45] Li J. Zuo J. Cai H. Zillante G. (2018). Construction waste reduction behavior of contractor employees: an extended theory of planned behavior model approach. J. Clean. Prod. 172, 1399–1408. doi: 10.1016/j.jclepro.2017.10.138

[B46] Lin B. Jia H. (2023). The role of peers in promoting energy conservation among Chinese university students. Human. Soc. Sci. Commun. 10, 1–10. doi: 10.1057/s41599-023-01682-2

[B47] López-Mosquera N. Lera-López F. Sánchez M. (2015). Key factors to explain recycling, car use, and environmentally responsible purchase behaviors: a comparative perspective. Resour. Conserv. Recycl. 99, 29–39. doi: 10.1016/j.resconrec.2015.03.007

[B48] Ma H. Chen Q. (2025). Social environment, low-carbon cognition and low-carbon consumption behaviors of youth groups: evidence from Xizang, China. Front. Psychol. 16:1494761. doi: 10.3389/fpsyg.2025.1494761 40012945 PMC11862823

[B49] Maduku D. K. (2024). How environmental concerns influence consumers' anticipated emotions towards sustainable consumption: the moderating role of regulatory focus. J. Retail. Consum. Serv. 76:103593. doi: 10.1016/j.jretconser.2023.103593

[B50] Masud M. M. Al-Amin A. Q. Junsheng H. Ahmed F. Yahaya S. R. Akhtar R. . (2016). Climate change issue and theory of planned behaviour: relationship by empirical evidence. J. Clean. Prod. 113, 613–623. doi: 10.1016/j.jclepro.2015.11.080

[B51] Meet R. K. Kundu N. Ahluwalia I. S. (2024). Does socio-demographic, greenwashing, and marketing mix factors influence Gen Z purchase intention towards environmentally friendly packaged drinks? Evidence from an emerging economy. J. Clean. Prod. 434:140357. doi: 10.1016/j.jclepro.2023.140357

[B52] Munir H. Jianfeng C. Ramzan S. (2019). Personality traits and theory of planned behavior comparison of entrepreneurial intentions between an emerging economy and a developing country. Int. J. Entrepreneurial Behav. Res. 25, 554–580. doi: 10.1108/IJEBR-05-2018-0336

[B53] Nawaz S. M. N. Alvi S. Rehman A. Riaz T. (2022). How do beliefs and attitudes of people influence energy conservation behavior in Pakistan? Heliyon 8:e10790. doi: 10.1016/j.heliyon.2022.e11054 36281414 PMC9586891

[B54] Nie J. J. Kou H. (2021). A study on influence of physical activity according to exercise attitude of adolescents. Korean J. Parent Educ. 18, 27–53. doi: 10.61400/JPE.2021.18.4.27

[B55] Niemiec R. M. Champine V. Vaske J. J. Mertens A. (2020). Does the impact of norms vary by type of norm and type of conservation behavior? A meta-analysis. Soc. Nat. Resour. 33, 1024–1040. doi: 10.1080/08941920.2020.1729912

[B56] Nitu-Antonie R. D. Feder E. S. (2017). Exploratory study on student's entrepreneurial intentions in developed and emerging countries. Rev. Manage. Comparat Int. 18:31.

[B57] Obradovich N. Migliorini R. Paulus M. P. Rahwan I. (2018). Empirical evidence of mental health risks posed by climate change. Proc. Nat. Acad. Sci. U.S.A. 115, 10953–10958. doi: 10.1073/pnas.1801528115 30297424 PMC6205461

[B58] Ogiemwonyi O. Harun A. B. Alam M. N. Karim A. M. Tabash M. I. Hossain M. I. . (2020). Green product as a means of expressing green behaviour: a cross-cultural empirical evidence from Malaysia and Nigeria. Environ. Technol. Innov. 20:101055. doi: 10.1016/j.eti.2020.101055

[B59] Ogiemwonyi O. Jan M. T. (2023). The influence of collectivism on consumer responses to green behavior. Business Strategy Dev. 6:542–556. doi: 10.1002/bsd2.261

[B60] Oludoye O. O. Supakata N. Srithongouthai S. Kanokkantapong V. Van den Broucke S. Ogunyebi L. . (2024). Pro-environmental behavior regarding single-use plastics reduction in urban–rural communities of Thailand: implications for public policy. Sci. Rep. 14:4713. doi: 10.1038/s41598-024-55192-5 38413669 PMC10899209

[B61] Paul J. Modi A. Patel J. (2016). Predicting green product consumption using theory of planned behavior and reasoned action. J. Retail. Consum. Serv. 29, 123–134. doi: 10.1016/j.jretconser.2015.11.006

[B62] Phuphisith S. Kurisu K. Hanaki K. (2020). A comparison of the practices and influential factors of pro-environmental behaviors in three Asian megacities: Bangkok, Tokyo, and Seoul. J. Clean. Prod. 253:119882. doi: 10.1016/j.jclepro.2019.119882

[B63] Pinho M. (2025). Does behaving green influence how we feel? pro-environmental behaviour and subjective well-being. BMC Psychol 13:1221. doi: 10.1186/s40359-025-03442-0 41189014 PMC12584498

[B64] Podsakoff P. M. MacKenzie S. B. Lee J. Y. Podsakoff N. P. (2003). Common method biases in behavioral research: a critical review of the literature and recommended remedies. J. Appl. Psychol. 88:879. doi: 10.1037/0021-9010.88.5.879 14516251

[B65] Podsakoff P. M. MacKenzie S. B. Podsakoff N. P. (2012). Sources of method bias in social science research and recommendations on how to control it. Annu. Rev. Psychol. 63, 539–569. doi: 10.1146/annurev-psych-120710-100452 21838546

[B66] Podsakoff P. M. Organ D. W. (1986). Self reports in organizational research: problems and prospects. J. Manage. 12, 531–544. doi: 10.1177/014920638601200408

[B67] Pontes S. Naranjo-Zolotov M. Painho M. (2024). From intention to action: how environmental setback perception mediates green purchase behavior. J. Clean. Prod. 470:143285. doi: 10.1016/j.jclepro.2024.143285

[B68] Qalati S. A. Qureshi N. A. Ostic D. Sulaiman M. A. B. A. (2022). An extension of the theory of planned behavior to understand factors influencing Pakistani households' energy-saving intentions and behavior: a mediated–moderated model. Energy Efficiency 15:40. doi: 10.1007/s12053-022-10050-z 35966953 PMC9362060

[B69] Ru X. Wang S. Chen Q. Yan S. (2018). Exploring the interaction effects of norms and attitudes on green travel intention: an empirical study in eastern China. J. Clean. Prod. 197, 1317–1327. doi: 10.1016/j.jclepro.2018.06.293

[B70] Ruangkanjanases A. You J. J. Chien S. W. Ma Y. Chen S. C. Chao L. C. (2020). Elucidating the effect of antecedents on consumers' green purchase intention: an extension of the theory of planned behavior. Front. Psychol. 11:1433. doi: 10.3389/fpsyg.2020.01433 32793023 PMC7393215

[B71] Saari U. A. Damberg S. Frömbling L. Ringle C. M. (2021). Sustainable consumption behavior of Europeans: the influence of environmental knowledge and risk perception on environmental concern and behavioral intention. Ecol. Econ. 189:107155. doi: 10.1016/j.ecolecon.2021.107155

[B72] Sabol M. Hair J. Cepeda G. Roldán J. L. Chong A. Y. L. (2023). PLS-SEM in information systems: seizing the opportunity and marching ahead full speed to adopt methodological updates. Indus. Manage. Data Syst. 123, 2997–3017. doi: 10.1108/IMDS-07-2023-0429

[B73] Schreinemachers P. Grovermann C. Praneetvatakul S. Heng P. Nguyen T. T. L. Sun Y. . (2023). Understanding consumers' purchase intentions of single-use plastic products. Front. Psychol. 14:1105959. doi: 10.3389/fpsyg.2023.1105959 36895736 PMC9988906

[B74] Shaw D. Kuo Y. L. Chie B. T. Chang C.-T. Hung M.-F. Chen H.-H. . (2025). Does the future imagination treatment affect people's pro-environmental intention and donation decisions? Environ. Dev. Sustain. 27, 1–21. doi: 10.1007/s10668-025-05967-1

[B75] Shimbar A. (2021). Environment-related stranded assets: an agenda for research into value destruction within carbon-intensive sectors in response to environmental concerns. Renew. Sustain. Energy Rev. 144:111010. doi: 10.1016/j.rser.2021.111010

[B76] Sun Y. He H. (2023). Understanding consumers' purchase intentions of single-use plastic products. Front. Psychol. 14:1105959. doi: 10.3389/fpsyg.2023.1105959 36895736 PMC9988906

[B77] Talwar S. Jabeen F. Tandon A. Sakashita M. Dhir A. (2021). What drives willingness to purchase and stated buying behavior toward organic food? A stimulus–organism–behavior–consequence (SOBC) perspective. J. Clean. Prod. 293:125882 doi: 10.1016/j.jclepro.2021.125882

[B78] Tam K. P. Chan H. W. (2017). Environmental concern has a weaker association with pro-environmental behavior in some societies than others: a cross-cultural psychology perspective. J. Environ. Psychol. 53, 213–223. doi: 10.1016/j.jenvp.2017.09.001

[B79] Tan Y. Wen Z. Hu Y. Zeng X. Kosajan V. Yin G. . (2023). Single-use plastic bag alternatives result in higher environmental impacts: multi-regional analysis in countries with uneven waste management. Waste Manage. 171, 281–291. doi: 10.1016/j.wasman.2023.08.040 37690403

[B80] Teraa S. Bencherif M. (2022). From hygrothermal adaptation of endemic plants to meteorosensitive biomimetic architecture: case of Mediterranean biodiversity hotspot in Northeastern Algeria. Environ. Dev. Sustain. 24, 10876–10901. doi: 10.1007/s10668-021-01887-y 34744498 PMC8563361

[B81] Tommasetti A. Singer P. Troisi O. Maione G. (2018). Extended theory of planned behavior (ETPB): investigating customers' perception of restaurants' sustainability by testing a structural equation model. Sustainability 10:2580. doi: 10.3390/su10072580

[B82] United Nations (2015). United Nations Transforming our World: The 2030 Agenda for Sustainable Development. New York, NY: United Nations General Assembly.

[B83] Van de Vijver F. Leung K. (1997). “Methods and data analysis of comparative research,” in Handbook of Cross-Cultural Psychology: Theory and Method, 2nd Edn. eds. J. W. Berry, Y. H. Poortinga, and J. Pandey (Allyn & Bacon), 257–300.

[B84] Venhof V. S. Jeronimus B. F. (2026). Emotional needs in the face of climate change and barriers for pro-environmental behaviour in Dutch young adults: a qualitative exploration. Int. J. Environ. Res. Public Health 23:76. doi: 10.3390/ijerph23010076 41595870 PMC12841537

[B85] Vorobeva D. Scott I. J. Oliveira T. Neto M. (2023). Leveraging technology for waste sustainability: understanding the adoption of a new waste management system. Sustain. Environ. Res. 33:12. doi: 10.1186/s42834-023-00174-x

[B86] Walton T. Austin D. M. (2011). Pro-environmental behavior in an urban social structural context. Sociol. Spectrum 31, 260–287. doi: 10.1080/02732173.2011.557037

[B87] Wang B. Yang R. Bai P. Fang Q. Jiang X. (2024). The plastic-reduction behavior of Chinese residents: survey, model, and impact factors. Sustainability 16:6093. doi: 10.3390/su16146093

[B88] Wang S. Wang J. Yang S. Li J. Zhou K. (2020). From intention to behavior: comprehending residents' waste sorting intention and behavior formation process. Waste Manage. 113, 41–50. doi: 10.1016/j.wasman.2020.05.031 32505110

[B89] Wang S. Yin N. Yang Z. (2021). Factors affecting sustained adoption of irrigation water-saving technologies in groundwater over-exploited areas in the North China Plain. Environ. Dev. Sustain. 23, 10528–10546. doi: 10.1007/s10668-020-01071-8

[B90] Warneke C. Schwarz J. P. Dibb J. Kalashnikova O. Frost G. Al-Saad J. . (2023). Fire influence on regional to global environments and air quality (FIREX-AQ). J. Geophys. Res. Atmospheres 128:e2022JD037758. doi: 10.1029/2022JD037758

[B91] Wu L. Y. Mu Y. F. Guo X. X. Zhang W. Zhang Z. M. Zhang M. . (2019). Encapsulating perovskite quantum dots in iron-based metal-organic frameworks (MOFs) for efficient photocatalytic CO_2_ reduction. Angew. Chem. Int. Ed. 58, 9491–9495. doi: 10.1002/anie.201904743 31066965

[B92] Yadav R. Pathak G. S. (2017). Determinants of consumers' green purchase behavior in a developing nation: applying and extending the theory of planned behavior. Ecol. Econ. 134, 114–122. doi: 10.1016/j.ecolecon.2016.12.019

[B93] Yuriev A. Dahmen M. Paillé P. Boiral O. Guillaumie L. (2020). Pro-environmental behaviors through the lens of the theory of planned behavior: a scoping review. Resour. Conserv. Recycl. 155:104660. doi: 10.1016/j.resconrec.2019.104660

[B94] Zhang S. Luo Y. Zhang P. (2024). A comparative study of factors influencing residents' waste sorting behavior in urban and rural areas of China. Heliyon 10:e30591. doi: 10.1016/j.heliyon.2024.e30591 38756576 PMC11096745

[B95] Zhang Y. Du J. Boamah K. B. (2023). Green climate and pro-environmental behavior: addressing attitude-behavior gaps towards promoting sustainable development. Sustain. Dev. 31, 2428–2445. doi: 10.1002/sd.2520

[B96] Zhang Y. Xiao C. Zhou G. (2020). Willingness to pay a price premium for energy-saving appliances: role of perceived value and energy efficiency labeling. J. Clean. Prod. 242:118555. doi: 10.1016/j.jclepro.2019.118555

